# The effect of multi-strain probiotics as feed additives on performance, immunity, expression of nutrient transporter genes and gut morphometry in broiler chickens

**DOI:** 10.5713/ab.20.0749

**Published:** 2021-03-02

**Authors:** Avishek Biswas, Kapil Dev, Pramod K Tyagi, Asitbaran Mandal

**Affiliations:** 1 Avian Nutrition and Feed Technology Division, ICAR-Central Avian Research Institute; Izatnagar, Bareilly, Uttar Pradesh 243122, India

**Keywords:** Antibiotic, Broiler, Gut Morphology, Immunity, Multi-strain Probiotic, Nutrient Transporter Genes

## Abstract

**Objective:**

This study was conducted to investigate the effects of dietary multi-strain probiotic (MSP) (*Bacillus coagulans* Unique IS2 + *Bacillus subtillis* UBBS14 + *Saccharomyces boulardii* Unique 28) on performance, gut morphology and expression of nutrient transporter related genes in broiler chickens.

**Methods:**

A total of 256 (4×8×8) day-old CARIBRO Vishal commercial broiler chicks of uniform body weight were randomly distributed into four treatments with 8 replicates each and having eight chicks in each replicate. Four dietary treatments were T_1_ (negative control-basal diet), T_2_ (positive control-antibiotic bacitracin methylene disalicylate at 20 mg/kg diet), T_3_ (MSP at 10^7^ colony-forming unit [CFU]/g feed), and T_4_ (MSP at 10^8^ CFU/g feed).

**Results:**

During 3 to 6 weeks and 0 to 6 weeks, the body weight gain increased significantly (p<0.05) in T_3_ and T_4_ groups. The feed intake significantly (p<0.05) reduced from T_1_ to T_3_ during 0 to 3 weeks and the feed conversion ratio also significantly (p<0.05) improved in T_3_ and T_4_ during 0 to 6 weeks. The humoral and cell mediated immune response and the weight of immune organs were also significantly (p<0.05) improved in T_3_ and T_4_. However, significant (p<0.05) dietary effects were observed on intestinal histo-morphometry of ileum in T_3_ followed by T_4_ and T_2_. At 14 d post hatch, the relative gene expression of glucose transporter (GLUT5), sodium-dependent glucose transporter (SGLT1) and peptide transporter (PepT1) showed a significant (p<0.05) up-regulating pattern in T_2_, T_3_, and T_4_. Whereas, at 21 d post hatch, the gene expression of SGLT1 and PepT1 was significantly (p<0.05) down-regulated in MSP supplemented treatments T_3_ and T_4_.

**Conclusion:**

The supplementation of MSP at 10^7^ CFU/g diet showed significant effects with improved performance, immune response, gut morphology and expression of nutrient transporter genes. Thus, the MSP could be a suitable alternative to antibiotic growth promoters in chicken diets.

## INTRODUCTION

Over many decades, poultry production in India and many other countries have had a spectacular growth explosion leading to a high profile industry. As 70% to 75% of the total costs of production are contributed by feed only, improvement of the feed conversion ratio (FCR) will significantly enhance the margin of profit. Antibiotic growth promoters (AGP) have been used widely to enhance the production capacity of poultry and protect them from pathogen risk. But due to the residual effect of antibiotics on human health, the use of anti-biotics in poultry feed is banned or going to be banned in many countries [1]. Potential alternatives to AGPs are therefore required to maintain health and use as growth stimulants in poultry. Moreover, there is concern about the side-effects of antibiotics uses as therapeutic agents, rise of antibiotic resistance and environmental pollution. The potential alternatives (probiotics, prebiotics, synbiotics, and postbiotic) to antibiotics that have been developed is in high demand for both consumers as well as and manufacturers. Nowadays, probiotics are being considered to be a suitable alternative, and therefore many progressive farmers in the world are incorporating them in poultry feed instead of antibiotics [2]. The use of probiotics is unavoidable to sustain poultry health at large-scale production units. After the ban of feed AGP in several countries, probiotics have garnered the lots of attention for poultry farmers as antibiotic usage has led to progress of antibiotic resistant microorganisms and the existence of antibiotic residues in animal products [3].

Probiotics are cultures of viable direct fed microbials which improve the health and productivity of broiler chicken [4] by immunomodulation and competitive exclusion of gut pathogens. Probiotics have been reported to improve the performance of chickens through sustaining a sound microbial equilibrium within the intestine to encourage the gut integrity and prevent enteric diseases [5]. Competitive exclusion, bacterial antagonism, and stimulation of the immune system are three key mechanisms of probiotics to improve the performance, immunity and gut health in chicken [6]. Probiotics develop resistance to pathogens, and subsequently decrease the pathogen load in gut which eventually improves the productivity index and immune status of the broiler chickens [7]. Chicken gastrointestinal tract (GIT) harbors diverse microbial community and their interactions significantly influence the nutritional, immunological and physiological status of the host [8]. Thus, the dietary modification of feed additives is a promising alternative which improves the overall gut health and immunity by fostering the growth of specific microbes [9]. In this regard, the dietary supplementation of probiotics is considered a promising alternative to AGPs [10]. Several studies have been piloted to conclude the effects of probiotics on growth performances and gut health of broiler chickens [11]. As the effects of probiotics are strain specific this study was conducted to investigate the effect of multi-strain probiotics (MSP) containing *Bacillus coagulans*-Unique IS2, *Bacillus subtilis* UBBS-14 and *Saccharomyces boulardii*-Unique-28 in the proportion of 2:2:1 respectively as feed additives on performance, immunity, gut morphometry and expression on nutrient transporter gene in broiler chickens.

## MATERIALS AND METHODS

### Ethics statement

The experimental procedures carried out in the study were approved by the Institutional Animal Ethics Committee (IAEC) (18 September 2017/Project No. 11) and guidelines of ‘Committee for the Purpose of Control and Supervision of Experiments on Animals (CPCSEA) 2012’ established under the ‘Prevention of Cruelty to Animals Act 1960’ of Indian Penal Code were followed.

### Supplements

The antibiotic bacitracin methylene di-salicylate (BMD) with 44% bacitracin activity was purchased from ALPHARMA Animal Health Division New Jersey-USA. The multi-strain probiotic-MSP containing *Bacillus coagulans*-Unique IS2, *Bacillus subtilis*-UBBS-14, and *Saccharomyces boulardii*-Unique-28 in the proportion of 2:2:1 respectively was obtained from Unique Biotech Ltd., Hyderabad, India. The MSP is is certified genetically safe as it doesn’t contain any putative virulence factors, antibiotic resistant genes and plasmids. The MSP used in this study are gram positive rods in the form of cream to brown coloured powder with water activity of less than one. Analysis confirmed absence of pathogens like *Escherichia coli*, Salmonella, Staphylococcus, and Pseudomonas absent in 10 g powder, and yeast mould count was not more than 100 colony-forming unit [CFU]/g.

### Birds, experimental design, diets and management

The experiment was conducted as per a completely randomized design. A total of 256 day-old straight run (sex ratio ≈ 1) commercial broiler chickens (CARIBRO-Vishal) of uniform body weight were randomly divided in to 32 replicate groups with 8 birds in each. The BMD, MSP was used in broiler chicken diets to formulated four dietary treatments *viz*. T_1_ (control diet), T_2_ (T_1_+20 mg antibiotic BMD/kg diet), T_3_ (T1+10^7^ CFU MSP/g feed), T_4_ (T_1_+10^8^ CFU MSP/g feed) respectively. The ingredient and nutrient composition of basal diet of broiler chicken is given in Table 1. The birds were vaccinated according to the routine vaccination programme followed at institute’s farm and provided *ad libitum* respective feed and fresh water throughout the feeding trial of 42 days. The birds were provided 24 h light on day one followed by a decrease of 1 h per day till it reached 18 h light period which was continued till the end of trial.

### Performance

Body weight gains (BWG) were recorded during the experimental period to determine the weekly and overall BWG. A weighed quantity of respective diet was offered *ad-libitum* daily to each dietary regimen in the morning and the residue was weighed next day on daily basis in order to arrive at overall feed intake (FI). Based on the data pertaining to the FI and BWG, the weekly and period wise FCR of birds was determined. Daily monitoring and recording on individual basis was carried out to study the mortality of the experimental birds used in the present investigation.

### Immune response

At 3 weeks of age, the immunization was done in broiler chickens. The antibody titer was determined by haemagglutination (HA) test methods [12,13] in U-bottom micro titer plate. Blood from jugular vein of healthy sheep was collected in Alsever’s solution. The blood was centrifuged at 2,500 rpm for about 10 minutes. The supernatant was discarded and the red blood cells were washed thrice in phosphate buffer saline (PBS). After washing, 1 mL of sheep red blood cells (SRBC) was added in 99 mL PBS to make 100 mL of 1% SRBC suspension and stored in refrigerator at 4°C until its use.

At 21st day post-hatch, 1.0 mL suspension of SRBC was injected intravenously to 24 birds per treatment (3 birds per replicates) to study the primary antibody response to SRBC. At 26th day (5 days post-immunization), 2 mL blood was collected from the wing vein. The blood was allowed to clot, the serum was collected, and frozen (−20°C) until analysed for the antibody titres to SRBC. At first, the microtitre plate was rinsed with 50 μL of PBS (pH 7.6) and 50 μL of sera was added in first well and, then 50 μL of 1% SRBC in PBS was added in each well and dried before the haem agglutination antibody (HA) titre was estimated by a micro-haem agglutination method [12] using two-fold serial dilutions of sera.

Cell mediated immune (CMI) response was assessed by cutaneous basophilic hypersensitivity test *in vivo* by using PHA-Pas per Corrier and Deloach [14], At 35 d of age, ten birds from each treatment were selected and the toe thickness of both left and right foot at 3rd and 4th inter digital spaces were measured by micrometer. Immediately after measurements 100 mg of PHA-P suspended in 1 mL of PBS and 0.2 mL of PBS was injected into right and left foot (acted as control), respectively. The web swelling of both the feet was measured 24 hours after injection by micrometer, as described by Cheng and Lamont [15]. The *in vivo* CMI response to PHA-P was expressed as Foot Web Index. Measurements made at 0 and 24 h after the injection, as described by Foot web swelling was calculated by subtracting skin thickness at 24 h post-injection from that at 0 h pre-injection.

### Carcass traits and cut up parts

Equal number of male and female birds was selected to avoid sex as a possible confounding factor. At the end of the experimental trial, four birds were selected randomly from each replicate of the treatment (32 birds per dietary treatment, n = 128) and sacrificed after 12 h of fasting with *ad libitum* drinking water for the assessment of carcass characteristics, organ weight and cut up parts.

### Expression of nutrient transporter genes

The jejunum tissue samples were collected aseptically in RNA later from five randomly selected birds from each treatment at 14 and 21 d post-hatching. The birds were sacrificed after stunning in an electrical water bath by severing the trachea and both carotid arteries. The expression analysis of Na+-D-glucose co-transporter 1 (*SGLT1*), fructose transporter (*GLUT5*), and H+-dependent oligopeptide transporter (*PepT1*) was performed in jejunum using glyceraldehyde 3-phosphate dehydrogenase (*GAPDH*) as housekeeping gene for normalization. Tissue samples were homogenized using an automated Kinematica polytron Homogenizier (Thermo Fisher Scientific, Gurgaon, Haryana, India) and total RNA was extracted from each jejunum tissues using Trizol (Invitrogen, Carlsbad, CA, USA) method according to the manufacturer’s instructions. The quantity and purity of RNA samples was analyzed by measuring the absorbance at 260 and 280 nm by using spectrophotometer (Nanodrop 1000, Thermo Fisher Scientific, Singapore). RNA integrity and purity was verified on 1.5% agarose gel by electrophoresis. The cDNA synthesis of RNA samples was carried out by using a “Revert Aid Revert Aid First Strand cDNA Synthesis Kit (MBI Fermentas, Hanover, MD, USA) by following manufacturer’s instructions. The quantity of cDNA samples was estimated by measuring the absorbance at OD 260/280 nm using Nanodrop 1000 (Nanodrop 1000, Thermo Fisher Scientific, Singapore) and cDNA samples were stored at −20°C for further use.

The cDNA samples were subjected to amplification by real-time quantitative polymerase chain reaction (PCR) using IQ5 Cycler system (Bio-Rad, Hercules, CA, USA). Amplification was carried out in 20 μL reaction containing quantitative PCR (qPCR) master mix of 1× SYBR GREEN dye (DyNAmo_HS; Finnzymes, Woburn, MA, USA), 0.2 μM concentration of 3′ and 5′ gene-specific primers (Table 2) and 2.5 μL of cDNA template. The qPCR conditions for 40 cycles were as follows: initial denaturation at 95°C for 15 min, subsequent denaturation at 95°C for 30 s, annealing at 60°C for 30 s, extension at 72°C for 30 s. The gene-related primers are listed in Table 2. All reactions were carried out in nuclease-free 8 tube-strips with optically clear flat caps (Axygen Scientific Inc, Union City, CA, USA). Results of amplification were expressed in terms of threshold cycle values (Ct), normalized against *GAPDH* gene, and fold expressions were determined by ^ΔΔ^CT method [16].

### Gut morphometry

At 21 and 42 days of age, ten birds per treatment (n = 10) were euthanized and jejunum tissue samples were collected. Two cross-sections were prepared on the glass slide for each sample of jejunum. One-cm segment of the midpoint of the ileum was removed, then washed the segments with physiological saline solution, and fixed in 10% buffered formalin. Each segment was then embedded in paraffin, and a 2 μm section of each sample was placed on a glass slide and stained with hematoxylin and eosin for examination. All the light microscopic variables were measured for jejunum of each bird using optical microscope (Motic Inverted microscope, Honkong), at a 4× magnification, a camera (Motic cam, CMOS, Honkong), and image analysis software (Motic Image 2.0, Honkong). The morphometric indices in each segment evaluated were villus height (VH), villus width (VW), crypt depth (CD), and the VH to CD. An average value was calculated for jejunum of each bird. The VH:CD ratio was then calculated accordingly.

### Statistical analysis

The experimental unit for data analysis was the sampled bird. Following a completely randomized design, the data were analysed by one way analysis of variance using the general linear model procedure (IBM SPSS softeware-20). The Tukey post-hoc analysis was done to test the significant mean differences between the groups with significance level defined at p<0.05.

## RESULTS

### Performance

The results of growth performance are shown in Table 3. During 3 to 6 weeks and 0 to 6 weeks of age the BWG of chickens increased significantly (p<0.05) in T_3_ and T_4_ compared to treatment T_1_ and T_2_, but the T_3_ was statistically similar to T_4_. The FI was significantly (p<0.05) reduced during 0 to 3 weeks and 0 to 6 weeks of age in T_3_ and T_4_ compared to other treatments T_1_ and T_2_, T_3_ was however, statistically similar to T_4_. The result indicated that FCR during 0–6 weeks of age significantly (p<0.01) improved in treatments T_3_ and T_4_ compared with T_1_ and T_2_.

### Immune response

The immune response of birds and weight of immune organs were significantly affected by dietary supplementation of MSP at 10^7^ or 10^8^ CFU per g diet (Table 4). Higher (p<0.01) index of humoral immunity and cell-mediated immunity were observed in treatment T_3_ followed by T_4_, T_2_, and T_1_. T_3_ was statistically similar to T_4_. The study revealed that the weight of immune related organs such as spleen (p<0.01) and thymus (p<0.05) was significantly affected in MSP incorporated treatment T_3_ and T_4_.

### Carcass traits and organ weight

Resulted parameters are presented in Tables 5 and 6. No significant difference was observed on carcass traits and different organ weights among the all dietary treatments. It was found that the weight of thigh, neck, breast and drumstick yield was significantly (p<0.01) higher in T_3_ followed by T_4_, T_2_, and T_1_, but T_3_ was statistically similar to T_4_.

### Gene expression

**Figure 1:**
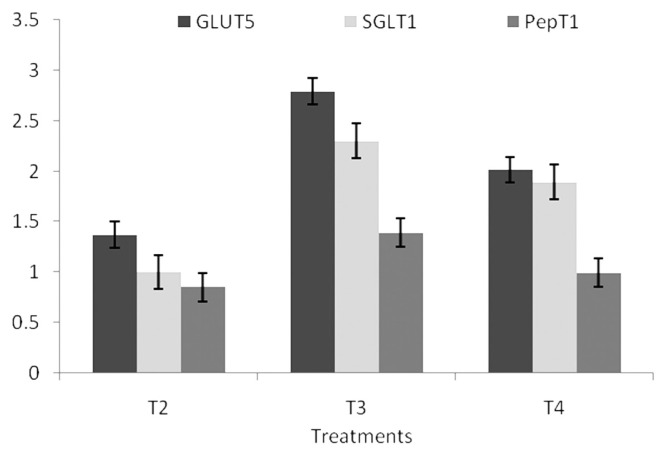
Effect of dietary MSP on GLUT5, SGLT1, and PepT1 expression in jejunum at 14 days in broiler chicken. MSP, multi-strain probiotic (*Bacillus coagulans*-Unique IS2, *Bacillus subtilis* UBBS-14, and *Saccharomyces boulardii*-Unique-28 in the proportion of 2:2:1, respectively); GLUT5, glucose transporter 5; SGLT1, sodium-dependent glucose transporter; PepT1, peptide transporter; CFU, colony-forming unit; BMD, bacitracin methylene di-salicylate. T1, control; T2, 20 mg BMD/kg; T3, MSP at 10^7^ CFU/g feed; T4, MSP at 10^8^ CFU/g feed.

**Figure 2:**
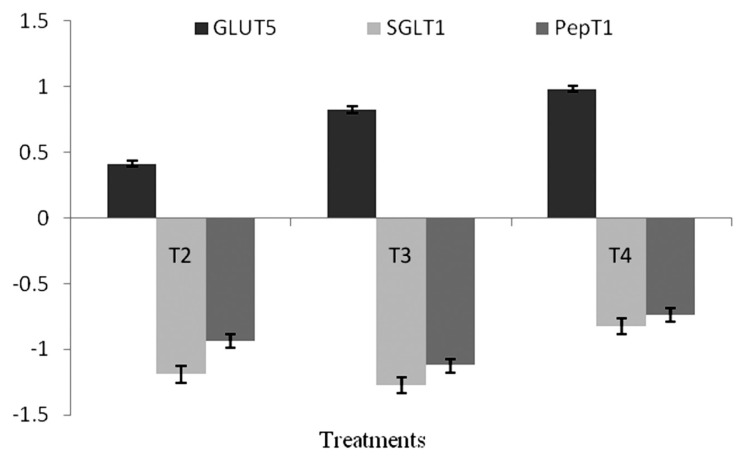
Effect of dietary MSP on GLUT5, SGLT1, and PepT1 expression in jejunum at 14 days in broiler chicken. MSP, multi-strain probiotic (*Bacillus coagulans*-Unique IS2, *Bacillus subtilis* UBBS-14, and *Saccharomyces boulardii*-Unique-28 in the proportion of 2:2:1, respectively); CFU, colony-forming unit; GLUT5, glucose transporter 5; SGLT1, sodium-dependent glucose transporter; PepT1, peptide transporter. T1 (control), T2 (20 mg BMD/kg), T3 (MSP at 10^7^ CFU/g feed), T4 (MSP at 10^8^ CFU/g feed).

This study observed that the expression of gene *GLUT5*, *SGLT1*, and *PepT1* was significantly (p<0.05) up-regulated in T_2_, T_3_, and T_4_ at 14 days of age (Figure 1). BMD and MSP supplemented groups did not differ significantly from each other. However, the expression of *GLUT5* gene was significantly up-regulated in T_4_ followed by T_3_, T_2_, whereas, significant (p<0.01) down regulation of *SGLT1* and *PepT1* gene was observed in T_3_ followed by T_2_ and T_4_ as compared to the control group (Figure 2).

### Gut morphology

**Figure 3:**
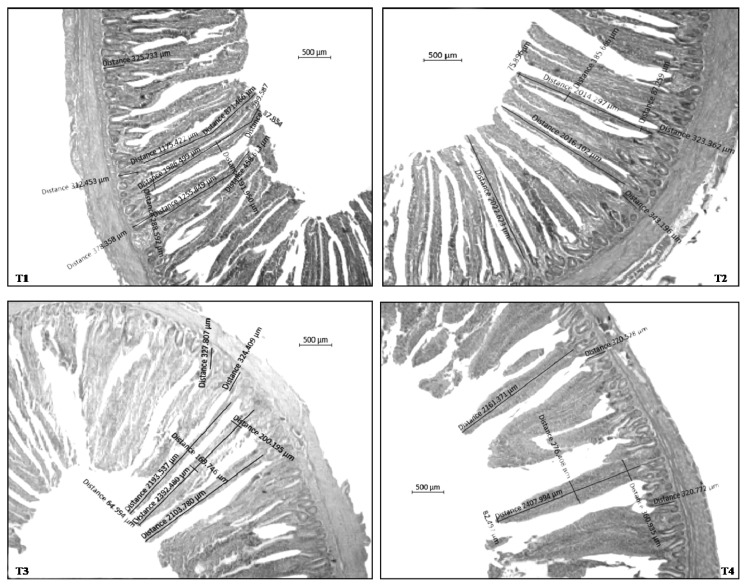
Effect of dietary MSP on small intestinal histo-morphology in ileum at 42 days in broiler chicken. MSP, multi-strain probiotic (*Bacillus coagulans*-Unique IS2, *Bacillus subtilis* UBBS-14, and *Saccharomyces boulardii*-Unique-28 in the proportion of 2:2:1, respectively); CFU, colony-forming unit. T1, control; T2, 20 mg BMD/kg; T3, MSP at 10^7^ CFU/g feed; T4, MSP at 10^8^ CFU/g feed.

The VH, VW, and CD were significantly increased (p<0.01) and ratio between villus height and crypt depth (VH:CD) were significantly improved (p<0.01) at 21 and 42 d in multi-strain probiotic supplemented groups i.e., T_3_ (10^7^ CFU MSP/m feed) and T_4_ (10^8^ CFU MSP/g feed) compared with other treated groups (Table 7; Figure 3).

## DISCUSSION

This study revealed that growth performance was improved by diets containing MSPs during 3 to 6 and 0 to 6 weeks of age compared with control and antibiotic treated group. This observation is similar to the study of Awad et al [17] who reported that the supplementation of probiotics with basal diet improved the BWG during the 3 to 6 weeks of birds. The results of the present study are however not in agreement with the findings of Junaid et al [18], and Chen et al [19]. Molnar et al [20] also reported that probiotic supplementation with basal diet improved the FI (0 to 3 weeks), but FCR (0 to 6 weeks) was consistent in both the growing (during 0 to 3 weeks of age) as well as finishing period (during 3 to 6 weeks of age). The overall performance and health of birds depends on many factors such as environmental stress, diet administration, farm sanitation, undefined microorganism and bird age [21]. However, the current study observed that the dietary MSP supplementation significantly (p<0.05) improved the growth performance during 0 to 3 weeks and 0 to 6 weeks of age of birds.

The result of the present study are in agreement with the findings of Lin et al [22] and Molnar et al [20] who also reported that anti-vaccine titre of probiotic treated birds were significantly higher than that of control birds. On the other hand, Lee et al [23] reported that no significant difference was observed in immune response after supplementing single strain direct fed microbial probiotic with basal diet. The present study revealed that dietary MSP supplementation had significant (p<0.05) effect on development of immunity related organ including spleen and thymus. Dietary probiotics may improve bird immunity through different ways-i) functioning as an agent and attaching to bacteria to initiate immune response, ii) direct promoting effect on immune system by active groups and competition with pathogen for nutrients and iii) inhibition of colonization of specific pathogen in gut of birds. The appearance of increased diffused lympho-histiocytic infiltration and solitary lymphoid follicles in the mucosa and a stronger response indicate increased immunological response in chicken fed with probiotic supplemented diets [18]. Yurong et al [24] reported that incorporation of probiotic in animal diet can stimulate the immune system by migrating through the intestinal wall as viable cells and multiply to a limited extent, causing production of immunogenic compounds, and mediating down-regulation of specific signalling pathways. Subsequently, stimulated immunity may manifest as enhanced macrophage activity and a systemic antibody response through enhanced production of immune-globulins (IgG, IgM), interferons, IgA levels at mucosal surfaces, and expression of various pro and anti-inflammatory cytokines [25,26]. The increase in the relative weight of the spleen and thymus by the groups supplemented with dietary MSP is consistent with observations of Paryad and Mahmoudi [27].

This study reported that the carcass traits (such as eviscerated yield and weight of heart, liver, and gizzard) did not significantly differ among the different treatments. The results of the present study are in agreement with Moreira et al [28] and Vargas Jr. et al [29] who also reported that no differences in carcass yield was observed between birds that were fed probiotics, antibiotic and control birds. Treatment with probiotic showed higher thigh, neck, breast and drumstick yield between control and treated groups except for back and wings (%). This observation is similar to what had been reported previously by Mokhtari et al [30]. On the other hand, Pelicano et al [31] observed no differences in cut up parts yield between control birds and those receiving single strain probiotics. The result of the present study are not in agreement with findings of Karaoglu and Durdag [32], and Raceviciute-Stupeliene et al [33], who reported no significant differences between in non-carcass component weights in control and treated group. In the study, measurement of some organ weights such as heart, liver and gizzard were determined but no significant differences were observed in non-carcass component weights. There was also no significant difference on overall carcass traits and organ weight in birds among the treatments. MSP incorporated in chicken diet influenced some carcass characteristics such as thigh, neck, breast, and drumsticks weights. This was in agreement with the findings of Wang et al [34] that probiotics have a growth promoting effect on cut up parts weights of chickens. It was also observed that MSP has no effects on abdominal fat which is contrary to the finding of Mohan et al [35] and Jin et al [36], who reported the reducing effect of single strain probiotics on fat deposition.

The jejunum is the key base of absorption in birds; therefore, the expression of nutrient transporters are responsible for dietary nutrient adjustment, impacts overall nutritional status, growth and development. The current study revealed that at 14 d post hatching, the expression of nutrient transporter genes was up-regulated due to BMD or MSP dietary supplementation. Whereas, at 21 d post-hatching, expression of only GLUT5 was up-regulated, SGLT1 and PepT1 in the BMD-supplemented group were down-regulated with respect to the MSP-supplemented groups. The major route for glucose assimilation in enterocytes is the SGLT1 transporter [37]. This may be why MSP supplementation outclassed BMD supplementation in terms of feed efficiency in the current study, thus making the importance of SGLT1 explicit in broiler growth performance. The absorption of di as well as tri-peptides occurs via proton-coupled PepT1 which is dependent on a pH gradient as well as a negative intracellular membrane potential [38]. Peptide transport by PepT1 is most efficient in an acidic environment [39], which is provided by MSP supplementation in broiler diets. However, the literature pertaining to the role of MSP supplementation in the nutrient transporter gene expression of broiler chicken is not available.

In the present study, MSP appear to influence the micro-structure of the gut more consistently. It affected VH, VW, CD, and the ratio of VH and CD in the ileum compared with control diets. This indicates that the absorptive function in the ileum of these chickens was higher compared with control treatments. The results of the present study are accordance with Iji et al [40] who found that, the ileal villi were significantly longer in chickens fed a control birds. The intestine can change its surface area by growing in length, and/or by increasing or decreasing the height of its villi when probiotics are supplied in the diet. Shortening and fusion of villi will result in loss of surface area for digestion and absorption of food [41], whereas the converse is true with longer villi and shallower crypts [42]. It is well-known that dietary probiotics lead to marked changes in the gut microflora, often favouring the host and the GIT has the ability to adapt by reacting morphologically to changing conditions such as altered diet [43]. It is a marker for gut health and can be evaluated by VH and CD [44]. The ileum is the major site for digestion and absorption of nutrients in the small intestine. Ileum histology, therefore was measured to monitor the expected negative effects of nitrogenous substances on VH. Shorter villi indicate a decrease in surface area for absorption of nutrients from the gut, as these structures are the functional areas for nutrient absorption [45]. An increase in height enhanced nutrient transport across the villus surface [46]. The shorter villi with greater CD in broilers fed antibiotic diet may be an indication of more damage to the gut by harmful compounds produced by microbial fermentation. A deeper crypt indicated increased turnover of enterocytes and, thus, more protein and energy demand for this purpose. The CD is an indicator of the number of crypt cells produced [47]. It has been reported that broilers spend approximately 12% of their synthesised protein on GIT turnover [48].

## CONCLUSION

The findings of this study evidenced that the presence of MSP at 10^7^ CFU/g feed had positive effect on performance, immunity and gut morphometry in broiler chickens. Cut up parts yield were also higher in the birds that received MSPs. This study also divulges that the MSP has potential for use as an alternative to antibiotics in broiler chickens diets; however, further study of this problem may be required to corroborate the evidence.

## Figures and Tables

**Table 1:** Ingredient and chemical composition of basal feed

Items	Starter (0 to 3 wk)	Finisher (4 to 6 wk)
Ingredients (%)		
Maize	54.005	60.005
Soya bean	39.600	33.600
Rapeseed meal	3.000	3.000
Limestone	0.900	0.900
Di-Calcium phosphate	1.700	1.700
Salt	0.300	0.300
DL-methionine	0.170	0.170
Lysine	0.010	0.010
TM premix 1^1)^	0.100	0.100
Vit premix 2^2)^	0.150	0.150
B Complex^3)^	0.015	0.015
Choline chloride	0.050	0.050
Chemical composition of basal diet		
Crude protein (g/kg)	223	200.6
ME (MJ/kg)	11.75	12.04
Calcium (g/kg)	10.9	10.9
Available P (g/kg)	5.0	4.2
Lysine (g/kg)	12.8	10.4
Methionine (g/kg)	5.1	4.3

ME, metabolizable energy.

1) Trace minerals (TM): premix (mg/kg) diet: MgSO_4_·5H_2_O, 300 mg/kg; MnSO_4_·H_2_O, 55 mg/kg; KI, 0.4 mg/kg; FeSO_4_·7H_2_O, 56 mg/kg; ZnSO_4_·7H_2_O, 30 mg/kg; CuSO_4_·5H_2_O, 4 mg/kg.

2) Vitamin premix supplied per kg diet: vitamin A (retinol), 8,250 IU; vitamin D_3_ (cholecalciferol), 1,200 IU; vitamin K (menadione), 1 mg.

3) B complex supplied per kg diet: vitamin B_1_ (thiamine), 2 mg; vitamin B_2_, 4 mg; vitamin B_2_ (riboflavin), 10 μg; niacin (nicotinic acid), 60 mg; pantothenic acid, 10 mg; choline, 500 mg.

**Table 2:** Nucleotide sequences of specific polymerase chain reaction primers

Gene^1)^	Primer sequence	Annealing Temp (°C)	Length (bp)	Gen Bank ID no.
*SGLT1*	F-TGTCTCTCTGGCAAGAACATGTC R-GGGCAAGAGCTTCAGGTATCC	60	71	XM_415247
*GLUT5*	F-TTGCTGGCTTTGGGTTGTG R-GGAGGTTGAGGGCCAAAGTC	60	60	XM_417596
*PepT1*	F-CCCCTGAGGAGGATCACTT R-CAAAAGAGCAGCAGCAACGA	60	66	NM_204365
*GAPDH*	F-GCCGTCCTCTCTGGCAAAG R-TGTAAACCATGTAGTTCAGATCGA	60	73	MN_204305

1) *SGLT1*, Na^+^-D-glucose co-transporter 1; *GLUT5*, Fructose transporter; *Pep T1*, H^+^-dependent oligopeptide transporter; *GAPDH*, glyceraldehyde 3-phosphate dehydrogenase.

**Table 3:** Effect of multi-strain probiotic on production performance of broiler chickens (0 to 6 wk)

Diet^1)^	Body wt gain (gm/bird)			Feed intake (gm/bird)			Feed conversion ratio			Mortality (%)
		
	0–3 wk	3–6 wk	0–6 wk	0–3 wk	3–6 wk	0–6 wk	0–3 wk	3–6 wk	0–6 wk	
T_1_	537.07	1,180.87^a^	1,717.94^a^	785.83^b^^c^	2,301.77	3087.6	1.46	1.95	1.80^ab^	2.08
T_2_	546.65	1,149.50^a^	1,696.15^a^	777.43^b^	2,359.69	3,137.12	1.42	2.05	1.85^b^	1.15
T_3_	544.81	1,235.29^b^	1,780.10^b^	742.39^a^	2,340.58	3,082.97	1.36	1.89	1.73^a^	0
T_4_	543.40	1,220.20^ab^	1,763.60^b^	738.14^a^	2,386.03	3,124.17	1.35	1.95	1.77^a^	0
SEM	2.04	6.85	8.27	7.83	13.56	19.31	0.02	0.06	0.04	-
p-value	0.135	0.021	0.001	0.022	0.072	0.083	0.110	0.063	0.008	-

SEM, standard error of the mean; BMD, bacitracin methylene di-salicylate; MSP, multi-strain probiotic (*Bacillus coagulans*-Unique IS2, *Bacillus subtilis* UBBS-14, and *Saccharomyces boulardii*-Unique-28 in the proportion of 2:2:1 respectively); CFU, colony-forming unit.

1) T_1_, control; T_2_, 20 mg BMD/kg; T_3_, MSP at 10^7^ CFU/g feed; T_4_, MSP at 10^8^ CFU/g feed.

a,b Mean values bearing the same superscripts in a column do not differ significantly.

**Table 4:** Effect of dietary multi-strain probiotic on immune response and relative immune organ (%) in broiler chickens

Diet^1)^	Immune response		Related immune organ (%)		
	
	Humoral (log_2_)	Cell mediated (mm)	Spleen	Bursa	Thymus
T_1_	1.79^a^	0.67^a^	0.19^a^	0.17	0.35^b^
T_2_	2.50^b^	0.74^ab^	0.20^ab^	0.15	0.33^ab^
T_3_	2.84^b^	0.99^b^	0.22^b^	0.16	0.34^ab^
T_4_	2.68^b^	0.91^b^	0.21^ab^	0.17	0.32^a^
SEM	0.092	0.062	0.03	0.01	0.03
p-value	0.006	0.000	0.005	0.075	0.015

SEM, standard error of the mean; BMD, bacitracin methylene di-salicylate; MSP, multi-strain probiotic (*Bacillus coagulans*-Unique IS2, *Bacillus subtilis* UBBS-14, and *Saccharomyces boulardii*-Unique-28 in the proportion of 2:2:1 respectively); CFU, colony-forming unit.

1) T_1_, control; T_2_, 20 mg BMD/kg; T_3_, MSP at 10^7^ CFU/g feed; T_4_, MSP at 10^8^ CFU/g feed.

a,b Mean values bearing the same superscript in a column did not differ significantly.

**Table 5:** Effect of dietary multi-strain probiotic on carcass traits and organ weight (% of live weight) in broiler chickens

Diet^1)^	Feather loss	Dressing yield	Eviscerated yield	Abdominal fat	Heart	Liver	Gizzard
T_1_	5.31	64.92	70.23	1.04	0.66	2.42	2.24
T_2_	5.36	65.04	70.24	1.03	0.65	2.37	2.17
T_3_	5.42	64.73	69.91	1.00	0.65	2.39	2.14
T_4_	5.59	64.79	69.87	1.08	0.66	2.30	2.12
SEM	1.89	4.36	5.56	0.04	0.02	0.08	0.09
p-value	0.255	0.123	0.089	0.072	0.113	0.118	0.068

SEM, standard error of the mean; BMD, bacitracin methylene di-salicylate; MSP, multi-strain probiotic (*Bacillus coagulans*-Unique IS2, *Bacillus subtilis* UBBS-14, and *Saccharomyces boulardii*-Unique-28 in the proportion of 2:2:1 respectively); CFU, colony-forming unit.

1) T_1_, control; T_2_, 20 mg BMD/kg; T_3_, MSP at 10^7^ CFU/g feed; T_4_, MSP at 10^8^ CFU/g feed.

**Table 6:** Effect of dietary multi-strain probiotic on different cut up parts (% of live weight) in broiler chickens

Diet^1)^	Thigh	Neck	Breast	Back	Wings	Drumstick
T_1_	9.81^a^	3.82^a^	16.93^a^	19.34	7.93	9.86^a^
T_2_	9.96^ab^	3.85^a^	16.94^a^	18.63	8.58	9.58^a^
T_3_	10.40^b^	4.01^b^	18.02^b^	19.75	9.09	10.26^b^
T_4_	9.74^a^	3.86^a^	16.68^a^	19.55	8.57	9.69^a^
SEM	1.89	0.42	4.36	3.56	1.05	1.27
p-value	0.015	0.002	0.000	0.072	0.111	0.003

SEM, standard error of the mean; BMD, bacitracin methylene di-salicylate; MSP, multi-strain probiotic (*Bacillus coagulans*-Unique IS2, *Bacillus subtilis* UBBS-14, and *Saccharomyces boulardii*-Unique-28 in the proportion of 2:2:1 respectively); CFU, colony-forming unit.

1) T_1_, control; T_2_, 20 mg BMD/kg; T_3_, MSP at 10^7^ CFU/g feed; T_4_, MSP at 10^8^ CFU/g feed.

a,b Mean values bearing the same superscript in a column did not differ significantly.

**Table 7:** Effect of dietary multi-strain probiotic on intestinal histo-morphometry in broiler chickens

Diet^1)^	Villus height (μm)	Villus width (μm)	Crypt depth (μm)	VH:CD
T_1_	2,086.1^a^	177.38^a^	260.8^a^	7.99
T_2_	2,089.7^a^	178.78^a^	270.2^a^	7.73
T_3_	2,456.8^b^	217.56^b^	321.3^b^	7.65
T_4_	2,245.9^ab^	199.06^ab^	312.0^b^	7.20
SEM	34.45	4.54	6.46	0.14
p-value	0.001	0.013	0.000	0.075

SEM, standard error of the mean; BMD, bacitracin methylene di-salicylate; MSP, multi-strain probiotic (*Bacillus coagulans*-Unique IS2, *Bacillus subtilis* UBBS-14, and *Saccharomyces boulardii*-Unique-28 in the proportion of 2:2:1 respectively); CFU, colony-forming unit.

1) T_1_, control; T_2_, 20 mg BMD/kg; T_3_, MSP at 10^7^ CFU/g feed; T_4_, MSP at 10^8^ CFU/g feed.

a–c Mean values bearing the same superscript in a column did not differ significantly.
